# Allergic sensitization: screening methods

**DOI:** 10.1186/2045-7022-4-13

**Published:** 2014-04-15

**Authors:** Gregory S Ladics, Jeremy Fry, Richard Goodman, Corinne Herouet-Guicheney, Karin Hoffmann-Sommergruber, Charlotte B Madsen, André Penninks, Anna Pomés, Erwin L Roggen, Joost Smit, Jean-Michel Wal

**Affiliations:** 1 DuPont Pioneer Agricultural Biotechnology, DuPont Experimental Station, 200 Powder Mill Road, Wilmington, DE 19880-0400, USA; 2 ProImmune Limited, The Magdalen Centre, The Oxford Science Park, Robert Robinson Avenue, Oxford OX4 4GA, United Kingdom; 3 Department of Food Science & Technology, Food Allergy Research and Resource Program, University of Nebraska–Lincoln, 143 Food Industry Complex, Lincoln, Nebraska, USA; 4 Bayer SAS, 355 rue Doistoïevski, 06903 Sophia Antipolis Cedex, France; 5 Department of Pathophysiology and Allergy Research, Medical University of Vienna, Waehringer Guertel 18-20, A-1090 Vienna, Austria; 6 Department of Toxicology and Risk Assessment, National Food Institute, Technical University of Denmark, 19, Mørkhøj Bygade, DK-2860 Søborg, Denmark; 7 TNO Triskelion BV, Utrechtseweg 48, 3700 AV Zeist, Netherlands; 8 Indoor Biotechnologies, Inc, 1216 Harris Street, Charlottesville, Virginia, USA; 9 Novozymes AS and 3Rs Management and Consultancy, Krogshoejvej 36, 2880 Bagsvaerd, Denmark; 10 Institute for Risk Assessment Sciences, Utrecht University, Yalelaan 104, 3508 TD Utrecht, Netherlands; 11 AgroParisTech, Department SVS, 16 rue Claude Bernard, F-75231, Paris Cedex 05, France

## Abstract

Experimental *in silico*, *in vitro*, and rodent models for screening and predicting protein sensitizing potential are discussed, including whether there is evidence of new sensitizations and allergies since the introduction of genetically modified crops in 1996, the importance of linear versus conformational epitopes, and protein families that become allergens. Some common challenges for predicting protein sensitization are addressed: (a) exposure routes; (b) frequency and dose of exposure; (c) dose-response relationships; (d) role of digestion, food processing, and the food matrix; (e) role of infection; (f) role of the gut microbiota; (g) influence of the structure and physicochemical properties of the protein; and (h) the genetic background and physiology of consumers. The consensus view is that sensitization screening models are not yet validated to definitively predict the *de novo* sensitizing potential of a novel protein. However, they would be extremely useful in the discovery and research phases of understanding the mechanisms of food allergy development, and may prove fruitful to provide information regarding potential allergenicity risk assessment of future products on a case by case basis. These data and findings were presented at a 2012 international symposium in Prague organized by the Protein Allergenicity Technical Committee of the International Life Sciences Institute’s Health and Environmental Sciences Institute.

## Introduction

In April 2012, an international symposium titled “Sensitizing Properties of Proteins” was held in Prague, Czech Republic, bringing together over 70 scientists from academia, government, and industry. The purpose of the symposium, organized by the Protein Allergenicity Technical Committee (PATC) of the International Life Sciences Institute’s (ILSI) Health and Environmental Sciences Institute (HESI), was to present data on the current state of the science regarding the sensitizing properties of proteins in relation to food allergy [1-3]. For the purposes of this manuscript, allergic sensitization is implied in the context of food allergy by the formation of antigen specific IgE.

Screening methods are the focus of this manuscript. Topics include *in silico*, *in vitro*, and rodent screening models that have been evaluated to predict protein sensitizing potential. In addition, background discussion is provided on whether there is evidence of new sensitizations and allergies since the introduction of GM crops in 1996, the importance of linear versus conformational epitopes, and protein families that become allergens.

### New proteins in the food chain: Is there evidence of new sensitization and allergies?

There is extensive knowledge of allergy to some foods and sensitization patterns (skin prick tests and specific IgE) to diverse dietary proteins. Scientists and the public often ask whether there is evidence that genetically modified (GM) crops have increased food allergy since they began entering the food chain in 1996 or whether people are becoming allergic to the novel proteins encoded by the inserted DNA [4]. Some people comment that the prevalence of food allergy is rising dramatically since the introduction of GM soybeans. However, the prevalence of food allergy to common specific food sources is low (*e.g.*, <0.01% for maize and approximately 0.4% for soybean) based on published reviews of studies using food challenges and detailed clinical histories [5,6]. Reports using only sensitization as measured by specific IgE tests have reported levels as high as 1 to 4% for the same foods [7]. Thus, claims of increased prevalence should be viewed with caution. Positive results with specific IgE and skin prick tests (SPT) do not prove allergy; rather, they prove only sensitization or cross-reactivity. More than 16 years after regulatory approval of the first GM plants, only one potential product was demonstrated to present a real risk of food allergy, *i.e.*, a soybean with a gene transferred from the Brazil-nut tree. The potential product was stopped during development and never commercialized, as testing with sera from Brazil-nut allergic subjects demonstrated IgE binding and SPT positivity for five Brazil-nut allergic subjects [8]. The evaluation was similar to the Codex Alimentarius Guideline strategy used to assess GM crop safety today [9].

The question of whether transformation of a gene might increase endogenous allergenicity has been raised. While there have been few controlled studies evaluating potential changes in endogenous allergenicity, a significant finding was widely differing variety-to-variety IgE binding to non-GM as well as GM varieties of soybean in a Danish study [10]. Studies at the University of Nebraska have evaluated IgE binding to three other GM soybean events by immunoblotting (1D and 2D) and ELISA inhibition, finding no significant differences between the GM and non-GM varieties [11].

Serum IgE binding to the CP4 EPSPS (5-enolpyruvylshikimate-3-phosphate synthase) protein in Monsanto’s herbicide tolerant soybean was tested as a post-market-monitoring evaluation using soybean allergic serum samples from Korea and central Europe [12]. No specific IgE binding was found to the CP4 EPSPS protein. Post-market-monitoring could be performed based on consumer complaint communication and follow-up, or by direct testing of selected populations. The intent of either is to sample the population of new consumers to measure sensitization rates and provide data for considering risk [13]. However, it is important to consider the technical challenges of measuring specific sensitization in real populations. The sample size and selection of subjects are critical, an estimate of exposure is essential, and baseline (pre-exposure) serum samples are helpful, as well as post-exposure measurement and clinical evaluation.

StarLink® maize, expressing approximately 50 ppm of Cry 9C protein from *Bacillus thuringiensis* that was toxic to various insect pests of maize and acts as a pesticide, was developed in the mid-1990s by Plant Genetic Systems of Belgium. The protein was stable to digestion in pepsin, a characteristic that is considered a potential risk factor for either sensitization or elicitation of food allergy. However, at such low concentrations, it is an unlikely candidate as a potential new food allergen, and there are a number of highly stable non-allergenic food proteins [14-16]. The product was not approved for food, and animal feed approvals were withdrawn even though no evidence of allergenicity was demonstrated in humans.

The alpha-amylase inhibitor (άAI) protein is expressed at up to 4% of protein in many rarely allergenic common beans (*Phaseolus vulgaris*). The gene was transferred into field peas (*Pisum sativum*) to protect the seeds from storage beetles which can cause 100% loss of product. The GM pea was tested in a non-validated animal model, triggering sensitization and eosinophilia during airway provocation [17]. The results have blocked further development of GM legumes with transferred άAI in regions that would certainly benefit from the products [9]. A more recent study was unable to reproduce the Prescott *et al.* findings, and reported that the GM pea was no more allergenic than non-GM peas and other legumes [18]. Evaluation of the άAI protein following Codex guidelines (unpublished, Goodman) indicated a need to test serum IgE binding with peanut sera due to low level sequence identity of άAI with peanut agglutinin, a protein rarely reported to cause allergy. Sera from 34 peanut allergic subjects failed to demonstrate cross-reactivity, but did uncover specific IgE binding to asparagine-linked carbohydrate determinants (CCD) on άAI in common with binding to CCD on other legume proteins [19]. However, basophil tests using the same sera failed to demonstrate activation, suggesting there is little likelihood of a risk of food allergy to this product. Certainly the risk is no different than that posed by common beans. As of 2013, there is no proof that the introduced protein in any approved GM plant has caused food allergy.

The introduction of whole new foods (*e.g.*, kiwifruit) in a population, or the introduction of commonly consumed foods in the diet of any individual, may lead to sensitization and food allergy. Although eating is a risk factor for food allergy, there are only certain foods that can cause significant food allergies, with only a few of the many proteins in food accounting for such allergic reactions. The Codex [20] guideline for evaluating food safety was designed to maximize the probability of identifying significant risks of food allergy. The primary risk would be introducing a major allergen from one source into a new food source or transferring a protein that is nearly identical to a major allergen and capable of causing cross-reactions. Those high risks are for individuals who are already sensitized, and methods for assessing such risks are relatively straightforward [9]. There is still much to learn about factors influencing sensitization and prediction of allergenicity; however, at this time, the most likely high-risk GM events can be identified and their introduction stopped.

### Allergenicity of linear versus conformational epitopes

Mapping studies of IgE antibody-binding epitopes have traditionally focused, by design, on the identification of linear epitopes by testing synthetic peptides or recombinant allergen fragments for IgE antibody recognition. However, the three-dimensional (3D) structure of the allergen has been ignored by this strategy, and information on conformational epitopes in allergens is lacking. Although conformational epitopes are common in inhaled allergens, food allergens may contain them as well if the allergen is not completely cleaved in the digestive tract and digestion-resistant fragments are absorbed. In recent years, conformational epitopes in seven allergens have been identified by solving the X-ray crystal structure of the allergen in complex with antibody fragments (Fab or Fab) [21]. This experimental approach is the most accurate way to define an epitope. Presently, there are no computational methods to accurately predict B-cell epitopes, and this experimental approach is the most accurate way to define an epitope. The antibodies used in these studies were either IgG that inhibit IgE antibody binding or recombinant IgE from combinatorial libraries made from blood of allergic donors. Allergens in complex with fragments of IgG antibodies recognizing epitopes that overlap with IgE antibody-binding sites have been reported for Api m 2, Bet v 1, Bla g 2, Der p 1, and Der f 1. At least four complexes of chicken egg lysozyme with IgG antibody fragments have also been reported, but the overlap of IgG and IgE epitopes is unknown. The structures of only two allergens (Bos d 5 and Phl p 2) in complex with recombinant IgE antibodies from combinatorial libraries have been solved. These studies have revealed mechanisms of allergen-antibody interaction and the structure of conformational epitopes. The nature of antibody recognition was defined by identifying key residues, as well as the kind of interactions involved (cation-π, hydrogen bonds, hydrophobic) [22-24]. Expression of epitope mutants in *Pichia pastoris* and antibody-binding analysis revealed the importance of mutated amino acids for the allergen-antibody interaction [23,24].

**Figure 1:**
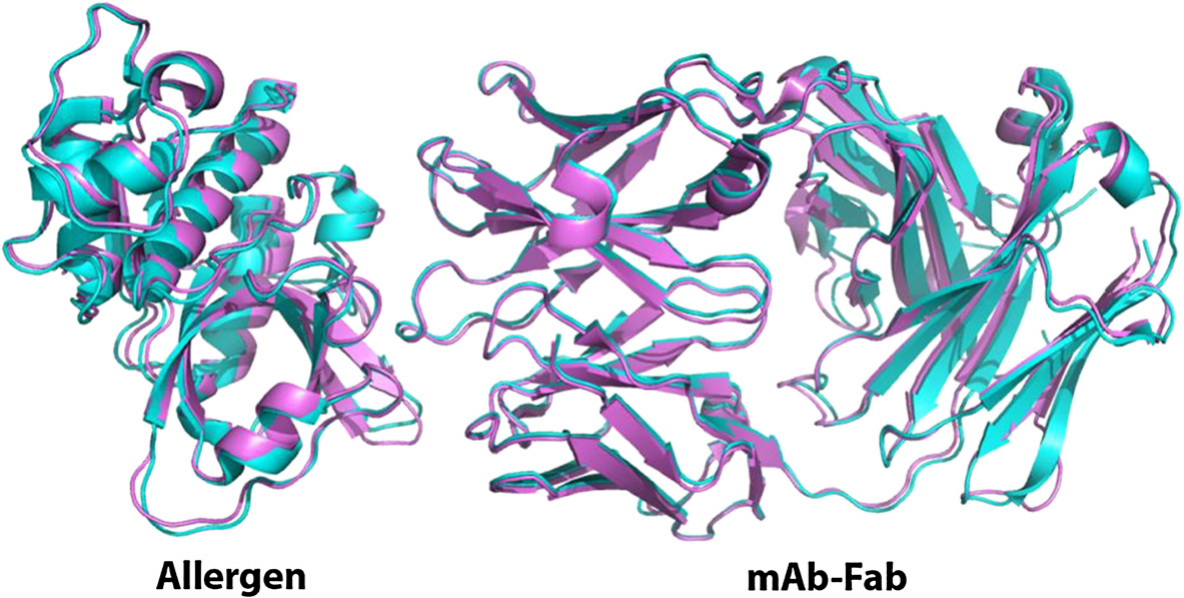
**Overlap of the X-ray crystal structures of natural Der p 1 in complex with an Fab of the mAb 4C1 (cyan) with the X-ray crystal structure of Der f 1 in complex with the same antibody fragment (violet).** The structures show the molecular basis of cross-reactivity between both dust mite allergens: the Fab recognizes the same area in Der p 1 and Der f 1, which is a conformational epitope overlapping with an IgE antibody binding site [24].

The structural basis of cross-reactivity between two allergens was reported for the first time by solving the structures of the dust mite allergens Der p 1 and Der f 1 in complex with a monoclonal antibody (mAb) that inhibits IgE antibody binding (Figure 1) [24]. Single mutants of residues in the epitope showed reduced IgE antibody binding, proving the overlap of the mAb epitope and IgE antibody-binding sites. Studies like these that take into account the 3D structure of allergens are also needed for food allergens to fully understand the B-cell repertoire and gain new insight into the molecular basis of cross-reactivity for allergenicity prediction [21].

The criteria for assessing risk of sensitization to new foods have evolved with time, precisely taking into account the fact that linear epitopes are not sufficient to explain the total IgE reactivity of proteins. The original guidelines for allergenicity assessment of GM crops used a decision tree based on few criteria [9,25,26]. One of them was the presence of small, contiguous identical amino acid matches to a known allergen in the sequence of the potentially allergenic protein as risk of cross-reactivity. This criterion resulted in a restricted identification of only potential linear epitopes. A slightly less conservative criterion of greater than 35% identity over 80 or more amino acids using FASTA or BLASTP was also proposed by the Food and Agriculture Organization (FAO) and the World Health Organization (WHO) of the United Nations panel, was accepted by the Codex Alimentarius Commission in 2003, and also acknowledged by the European Food Safety Authority (EFSA) [27-29]. This new criterion addressed the fact that the molecular basis of cross-reactivity is not only in the primary structure of the proteins, but in the tertiary structure as well. Therefore, it takes into account the existence of conformational B cell-epitopes. In general, just considering the primary structure of the protein, <50% amino acid identity among proteins rarely results in antigenic cross-reactivity. At this low level of identity, and for proteins with the same overall fold, the molecular surface is quite different. High risk of cross-reactivity exists among proteins with >70% identity [9,30]. Exceptions to this general rule may exist because, in addition to the primary structure of the allergen, the tertiary structure of the protein plays a major role in IgE antibody recognition. Nevertheless, the guidelines mentioned above are useful for assessing potential cross-reactivity among homologous proteins.

Antibodies recognize a group of residues from which only a few (approximately 5-8) contribute most of the binding energy and are the essential amino acids from conformational epitopes [31]. These residues are usually non-consecutive and located far apart in the primary structure of the protein, involving different loops or secondary elements of the allergen. Therefore, it is very useful to know the 3D structures of allergens in order to locate antibody recognition sites. Currently, there are approximately 715 allergens in the official database for the systematic allergen nomenclature that is approved by WHO and the International Union of Immunological Societies (WHO/IUIS) Allergen Nomenclature Sub-committee (http://www.allergen.org). However, the 3D structure has been solved only for ~75 allergens, *i.e.*, ~10% of the allergens in the database, from which only ~24 are food allergens (as assessed from the WHO/IUIS Allergen Nomenclature database and the Protein Data Bank). Although there is no simple rule or algorithm that allows prediction of protein allergenicity, information derived from the 3D structure of allergens and conformational epitopes is very useful to assess the allergenicity of new proteins on a case-by-case basis. Specifically, the location of IgE antibody-binding epitopes in allergens can be useful for the identification of potential allergens among novel allergen-homologous proteins.

### Protein families that become allergens

Only a restricted number of protein families accounts for the majority of food allergic reactions in predisposed individuals. In fact, only 2% of all known protein families so far contain allergenic proteins [32]. The most important allergenic protein families from plant foods are the prolamin superfamily, including the non-specific lipid transfer proteins (nsLTPs) and the 2S albumins, as well as the cupin superfamily with the 11S and 7S globulins [33]. nsLTPs are relevant plant food allergens with a robust structure due to four disulfide bridges, which makes them less susceptible to gastrointestinal digestion. Especially in fruits from the Rosaceae family, nsLTPs account for severe allergic reactions in patients. In addition, nsLTPs have been identified in pollen, tree nuts, peanut, and vegetables. Although the overall protein fold is present in all these nsLTPs, their cross-reactivity with clinical relevance is related to sequence similarity and follows the botanical relationship [34].

2S albumins also are proteins with a distinct robust 3D structure, formed by four disulfide bridges. 2S albumins from peanut and tree nuts are recognized as allergens that induce rather severe allergic reactions, while for other plant species (*e.g.*, sunflower 2S albumin), allergenic reactivity is weaker. Reports identified cross-reactivity among 2S albumins from peanut and lupine, mustard and rapeseed, and sesame and poppy seeds, respectively.

The 7/8S and 11S seed storage globulins are the major components of seeds of dicotyledonous species. Typical allergens from the 7/8S protein family are Ara h 1 from peanut and Jug r 2 from walnut. The 11S globulins are allergenic proteins in peanut (Ara h 3), soybean (Gly m 6) and Brazil nut (Ber e 2). Cupins share a similar overall fold but sequence similarities among proteins from different botanical families are low and thus cross-reactivity is limited.

The panallergen profilin and the Bet v 1-related proteins have been identified in various plant tissues and are mostly responsible for the pollen-food cross-reactivity. Profilins are ubiquitous proteins, involved in various cell signalling pathways such as cytokinesis. Despite rather high sequence similarity among different plant profilins (around 75%) and frequent IgE cross-reactivity, their clinical significance as allergens seems to be restricted to certain plant foods such as melon and citrus fruits. Usually pollen profilins are regarded as the primary sensitizers, and the protein displays an intermediate stability when subjected to gastro-duodenal digestion. However, regarding the sensitization patterns to profilins, a North-South difference in Europe could be identified with a higher relevance in Southern Europe versus a lower sensitization range in Northern parts of Europe [35]. Bet v 1 related proteins all share a similar 3D structure and display rather high sequence similarity. These proteins are identified as allergens in a range of pollens and plant foods usually evoking mild symptoms, whereas in some cases, as in soybean, the Bet v 1 homologous allergen accounts for severe reactions in patients.

Among the animal food allergens, parvalbumins, tropomyosins, and the caseins are the most important protein families. Parvalbumins are the major allergens in fish and are characterised by an EF hand motif. They are Ca^2+^ binding proteins (composed of two helixes, E and F) with resistance to heat treatment and proteolysis. Their characteristic fold contributes to the IgE binding activity and, together with sequence similarity, accounts for cross-reactivity with clinical significance. Tropomyosins, highly conserved proteins with a central intracellular function, are major allergens from crustaceans and molluscs. They are also identified as allergens in mites and cockroaches. Their alpha helical structure remains unaffected when subjected to heat treatment and enzymatic digestion. Caseins are proteins restricted to mammalian species and are abundant in milk. They bind Ca^2+^ and typically form clusters with a random coil structure. IgE cross-reactivity between caseins from different species is related to sequence similarity.

In summary, it seems that conserved structures and certain biological activities contribute to allergenic activity. Within a protein family, the presence of highly conserved surface structures and sequence identities above 50% account for clinically relevant cross-reactivity. Efforts have been undertaken to study the physicochemical properties of allergens and to identify relevant IgE-binding epitopes, which in turn helped to discriminate between hypo- and hyper-allergenic molecules. These well-defined proteins can now be used to study molecular mechanisms involved in their uptake across mucosal barriers and their interaction with the immune system [36]. Improved knowledge on the specific uptake and processing of allergens will contribute to understanding the factors that make a protein an allergen.

### Evaluation of the sensitizing potential of industrial enzymes using the results from Sens-it-iv, a project funded by EU Framework Programme 6

The number of industrial applications involving proteins has been increasing during the last decade. In particular, enzymes are increasingly used for processing food and as food additives for human and animal use. Along with these industrial applications, more and more new foods are being introduced, while established foods are genetically modified to contain new proteins providing them with new favorable characteristics. The use of proteins in applications intended for consumption exposes consumers to a potential risk for acquiring an allergic response. Strategies for risk assessment and risk management of novel proteins in food continue to evolve. Currently, risk assessment is based on amino acid sequence similarities of a novel protein with known allergens, gene source, physicochemical similarities, and when necessary (*i.e.*, a positive amino acid match to a known allergen), IgE binding studies with relevant allergic patient sera.

European legislation (*e.g.*, Directive 2012/69/EU) and the US National Research Council (NRC) vision and strategy for toxicity testing in the 21st century (Tox21) [37] are currently driving toxicity testing from animal-based testing towards *in vitro* testing using human cell-based tests addressing pathways of toxicological concern (*e.g.*, sensitization and allergy development [38,39]). While relevant pathways of toxicological concern have not yet been accurately described for sensitization and allergy triggered by food proteins, progress has been made in the understanding of skin sensitization to chemicals and respiratory sensitization to both chemicals and proteins [40,41].

**Figure 2:**
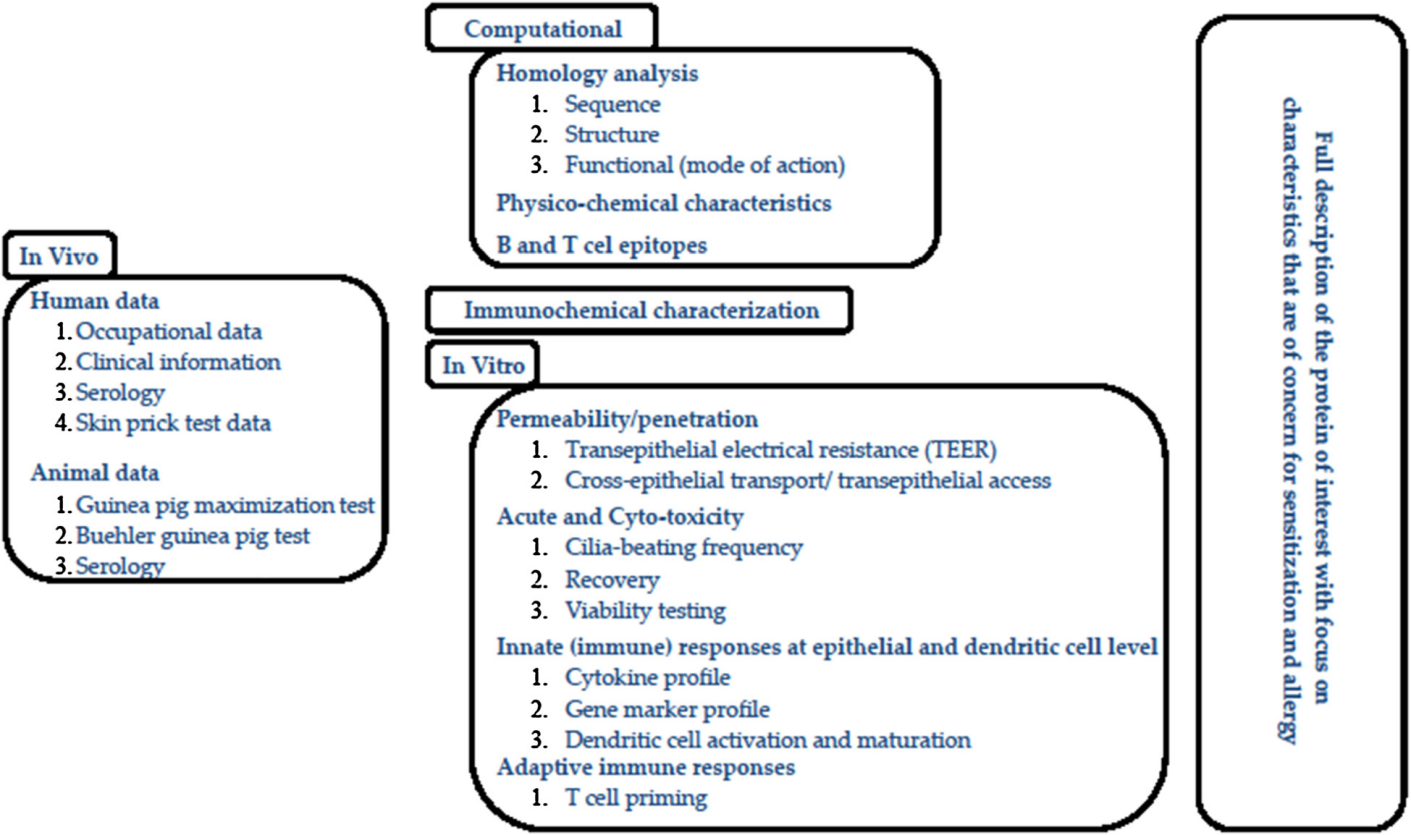
Testing strategy for determining the lung sensitizing capacity and relative potency of proteins.

Currently, selected tests from the Sens-it-iv toolbox (Table 1) are in the process of being implemented in strategies for assessing the relative risk associated with the production (occupational risk) and use of novel proteins in consumer product applications. A testing strategy for determining the lung sensitizing capacity and relative potency of serine proteases in an industrial setting (occupational risk) has been developed. Novel proteins are assessed relative to well-established industrial proteins for which historical *in vivo* and *in vitro* data are available and for which the exposure scenario has been established. The “benchmark” approach was preferred because the novel *in vitro* test approaches are not yet considered sufficiently documented for allowing assessment of the absolute risk associated with the application of a novel protein. The preferred strategy is a weight-of-evidence approach that compares in a descriptive, semi-quantitative way the impact of the benchmark protease and a novel protease on well-established pathways of toxicological concern (Figure 2). The initial step of the strategy is to collect all the *in vivo* and *in vitro* data that are available on enzymes going through the same production processes in general and of proteases specifically. These historical data are to be used as reference data for bridging the gap between human occupational safety and computational, immunochemical and *in vitro* data.

**Table 1:** The Sens-it-iv toolbox

Keratinocytes	NCTC2544 test
	Human reconstituted skin
Lung EC	Precision cut lung slices
	Human reconstituted *alveolar* epithelium
	Human reconstituted *bronchial* epithelium
	Specific sensitizer profil
DC	Xenobiotic sensing (genomic profile)
	Maturation #1 (CD86, CD54, IL-8, …)
	Maturation #2 (DotSCan)
	Migration
T-cells	Primary T-cell stimulation
Other	Neutrophil - THP-1 metabolization tests
	Proteomics marker profile (combined list)

Computer-aided data mining systems are extremely useful tools for identification of potential modes of action by which the protein of interest may cause sensitization and allergy. Several lines of evidence suggest that protease activity may facilitate sensitization, and several mechanisms have been postulated [40]. The acquired information makes it possible to optimize the *in vitro* testing strategy for characterization of the enzyme. Sequence and structural homology of the targeted protein with proteins that are known to be involved in allergic inflammation and lipid-binding can provide additional information about the potential risk for the protein being an allergen [42]. Methods for assessing similarities between primary amino acid sequences, physicochemical and structural propensities, and 3D structures of proteins with known and unknown antigenicity are used to predict the allergenic potential of a novel protein. However, comparison of the available technologies has revealed that antigenicity of a protein is not described by these characteristics alone. Molecular characteristics of the amino acid sequences involved in T- and B-cell epitopes are required [43]. T-cell epitope mapping studies and computational algorithms have already allowed the identification of several major T-cell epitopes, and are believed to refine the understanding of the immune responses to allergens [44]. There is no computational method available yet that can predict B-cell epitopes in a reliable way, though a few have been reported on a number of proteins [45].

Sera from exposed and non-exposed humans and animals (typically guinea pigs, mice, rats and rabbits) can be used in a direct or a competitive ELISA to assess to what extent a novel protein shares B-cell epitopes with the benchmark protein (cross-reactivity). It has to be stressed, however, that additional *in vivo* studies and immunochemical analyses have to be performed in order to eliminate the possibility that the novel protein has epitopes that are not present on the benchmark protein.

**Figure 3:**
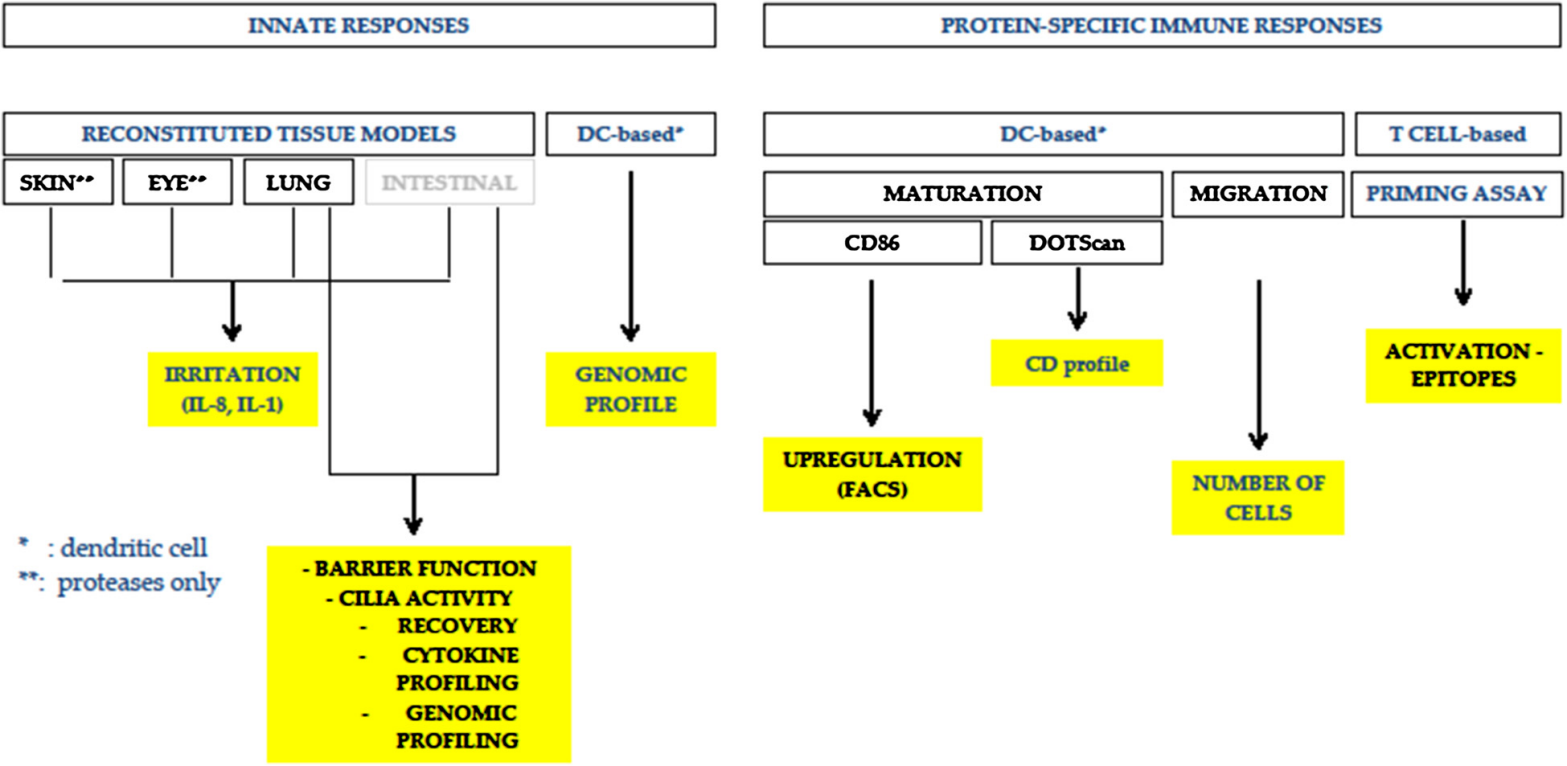
Testing strategy for determining the lung sensitizing capacity and relative potency of proteins.

A variety of *in vitro* tests addressing specific key events of sensitization are now available for the skin and the respiratory tract. Figure 3 suggests a strategy addressing different aspects of sensitization. It is now generally accepted that for a compound to trigger sensitization, it must have the capacity to (i) penetrate the tissue, (ii) trigger inflammation at both the epithelial and dendritic cell (DC) level thereby creating the micro-environment resulting in (iii) DC activation and migration to the lymphoid tissue, and finally (iv) T-cell priming. The availability of reconstituted human alveolar and bronchial tissue models makes it possible to assess how easy it is for a protease to gain access to critical cells of the innate and adaptive immune responses (*e.g.*, DCs, basophils). There is substantial evidence that correlates easy access with allergenicity [40].

There is growing evidence that sensitization through the skin and the lung is the result of an inflammation reaction that is driven by both epithelial cells and DCs [40,46]. Currently, cytokine profiles describing respiratory sensitizers (chemicals and proteins) are being evaluated. The current status is that cytokine profiles have been identified that relate to different enzyme activities (proteases, lipases, amylases), but there is evidence that a common set of cytokines may exist. For proteases, a specific macrophage colony-stimulating factor seems to be directly related to the allergenic potential of the protease. The test also indicates that the family of serine proteases contains members that differ substantially in their allergenic potency. This observation was confirmed by preliminary studies in mice. In addition to the epithelial cytokine profiles, a DC-derived marker gene profile is under evaluation.

DC activation and migration are the result of a proper inflammatory response. The Sens-it-iv project has developed tests addressing these phenomena. DC activation can be assessed by quantifying the expression levels of specific markers, such as CD54, CD80, and CD86, or by describing a CD profile using, *e.g.*, the DotScan approach. The potential of a protein to trigger cell migration is assessed in a two-compartment test format [47]. The ultimate test describing the potential of a protein to cause sensitization is the *in vitro* T-cell priming assay [48]. Since this test requires fresh blood samples from human volunteers, donor-to-donor variability is an important issue. Nevertheless, it has been shown that the test can provide useful information about the allergenic potential of proteins.

Collecting the information related to specific events known to be important for sensitization is one side of the story; integrating the data and coming to a conclusion about the relative risk of a protein is the other side. Table 2 provides an overview of the information that has been or is in the process of being collected for proteases (N = 3), amylases (N = 2), and lipases (N = 1). The current strategy is to assign weights to the various test results based primarily on mechanistic considerations. Thus, the more protease required for disturbing the epithelial barrier (quantified by transepithelial electrical resistance and viability measurements) or inducing macrophage colony-stimulating factor expression, the more likely it is that it will penetrate and cause sensitization. The weight for this observation could be defined for example by means of the amount of protein required to induce a 50% change in the measured parameter.

**Table 2:** Data integration

**Enzyme activity**	**Variant/ class**	***In vivo *data**		**Computational**		**Innate immune responses**					**Adaptive immune responses**		
		Animal	Human	B cell	T cell	Barrier function (mg/ml)	Cilia beating (mg/ml)	Recovery	Cytokine profile (mg/ml)	Genomic profile	DC maturation	DC migration	T cell priming
Protease	1	**Animals**		Epitope lists available		0.01	0.01	no	0.001	Analysis in progress	Analysis in progress		Epitopes identified
		Guinea pig, Rat, Mouse Serological data											
				Overlaps and differences identified									
	2					0.1	0.1	no	0.1				
	3					10	10	yes	10				
		Immunochemical characterization											
Amylase	A			Epitope lists available		10	10	yes					
	B					No effect	No effect	--					
				Overlaps and differences identified									
		**Humans**											
		Clinical studies, Occupational data											
		Serological data											

Others (Lipase)				Epitope lists available		No effect	No effect	--					

It is not clear yet how to interpret partial cross-reactivity, reduced affinity for specific antibodies, and small differences between observed T- and B-cell epitopes. When no cross-reactivity between the benchmark protein and the novel protein is observed or the identified epitopes are identical for both proteins, the message is rather clear. However, experimental data suggest that epitopes may differ in 1 or 2 amino acids without loss of affinity for antibody binding (Roggen, personal communication). Based on the acquired data, it is obvious that the term “significant similarity” which is so often used in the context of the assessment of protein allergenicity still remains to be defined properly.

The computational, immunochemical and *in vitro* approaches are to provide a patchwork of data where each patch addresses a specific potentially hazardous event. It is anticipated that an intelligent combination or addition of these individual hazards will allow a qualified overall hazard identification of the novel protein as compared to the benchmark. In order to move from hazard identification to risk assessment, an in depth knowledge of the potential *in vivo* exposure routes as well as exposure levels is required. In an occupational setting, such knowledge rarely exists. Exposure levels are established by means of cross-sectional studies among eligible workers of a production plant. They are interviewed for respiratory and allergic symptoms, blood samples are taken to examine sensitization to enzymes, and a clinical evaluation is performed. In addition, exposure in the plant is characterized qualitatively, and exposure levels are estimated semi-quantitatively. Finally, workers are classified into exposure groups with varying exposure profiles to enzymes, based on frequency, duration, and anticipated level of exposure [49]. Due to the uncertainty of the actual exposure levels, no-effect levels are defined empirically on the basis of recorded cases of sensitization at the production plant and tend to be conservative. These uncertainties should be taken into consideration when *in vivo* human data are used as references during the development of *in vitro* tests and non-animal based testing strategies.

This summary describes a means to evaluate the risk of industrial proteins, such as proteases, to cause occupational sensitization. At this point, the tool is at an early stage of development and various questions remain to be answered. Tests addressing the key events of skin and respiratory sensitization are available. The question remains whether all of these tests have to be implemented or if there is a minimal number of (simple) tests that would be equally predictive. For skin sensitization, there is increasing evidence that two or three tests addressing specific innate responses provide a 96% concordance with the available *in vivo* data. Could this also be sufficient for assessing novel proteins? It is not yet clear how the various data should be integrated, nor how the *in vivo* information should be used to bridge the gap between non-animal testing and assessment of human safety. The weight of each input still has to be established. Finally, acquiring sufficient reliable information about the exposure routes and levels will constitute an important obstacle for the current strategy to become a tool for risk assessment and risk management. In spite of these hurdles, the goal in the near future is to be able to perform “relative” hazard identification and risk assessment of novel industrial enzymes. Furthermore, it is believed that the learnings from industrial enzymes will be helpful in establishing similar testing strategies for novel food proteins and biopharmaceuticals.

### The mouse cholera toxin model in translational testing of allergenicity of proteins: from *in vivo* to *in vitro*

Without doubt, animal models have contributed insight into the mechanisms of sensitization to food proteins and development of food allergy. In mouse models, the mucosal adjuvant cholera toxin (CT) is generally used to induce allergic sensitization to co-administered proteins in mice, while feeding the protein alone induces oral tolerance. CT induces innate immune changes that trigger allergen-specific T- and B-cell responses, leading to an allergic phenotype. These innate immune changes involve activation of epithelial cells (ECs), intraepithelial lymphocytes (IELs), DCs, and induction of co-stimulatory molecules [50-52]. Understanding the mechanisms by which CT can break mucosal tolerance is of interest as the same pathways may be playing a role in human food allergy. To provide a mechanism for allergic sensitization in the intestine, it was hypothesized that molecular stress imposed on gut epithelial cells, for instance by CT or non-steroidal anti-inflammatory drugs, is a principal trigger for ECs and IELs to subsequently activate DCs and T and B cells. To explore this hypothesis, IELs were isolated from mouse intestine and investigated for their interaction with a mouse intestinal epithelial cell line (MODE-K) and with stem cell-derived intestinal organoids. The latter consist of epithelial crypts, containing enterocytes, Paneth cells, goblet cells, and endocrine cells [53]. Co-culture of MODE-K cells and IELs in the presence of CT results in cytokine secretion of MODE-K, increased degranulation of IELs, and upregulation of MHC II on both cell types. These *in vitro* co-culture systems might contribute to further understanding of early mechanisms of sensitization. Together with specific animal models, such *in vitro* models may eventually help to assess allergenicity of novel proteins. Other experiments have shown that intestinal sensitization (using CT) involved the disappearance of IELs in the intestine, possibly resulting in a loss of tolerogenic signals in the intestine leading to sensitization. Indeed, following blockade of IELs, allergic sensitization to peanut was enhanced [51].

To assess allergenicity *in vivo*, it is important to realize that, although the principal allergic response in the cholera toxin allergy model is driven by CT, the properties of the administered protein may be even more decisive. In detailed studies in a peanut allergy mouse model, mechanisms of food allergy and anaphylaxis were investigated, along with the relative contribution of the individual peanut allergens to clinical food allergic responses. In three commonly used mouse strains (C3H, C57BL, and BALB/c), sensitization and challenge induced equal levels of specific IgE, IgG1, and IgG2a [54]. However, mast cell degranulation and anaphylactic symptoms after systemic challenge with peanut did not correlate. Most interesting was the occurrence of anaphylaxis in the absence of mucosal mast cell degranulation in C57BL/6 mice. However, using FcyR knock-out and mast cell-deficient mice, the anaphylaxis observed in C57BL mice was entirely dependent on FcyR and mast cells. In addition, the reported anaphylaxis in C57BL mice, unlike in C3H mice, was partially dependent on platelet activating factor (PAF) and macrophages. This indicates that the allergic cascade, leading to clinical manifestations, differs between the three mouse strains. Moreover, it seems that clinical manifestations of food allergy in the mouse are not uniquely linked to the classical components of the allergic cascade but may also comprise the “alternative anaphylaxis pathway,” involving IgG, macrophages, and PAF. This might explain the pleiomorphic manifestations of food allergy in the human situation.

In addition, individual peanut allergens were found to differ significantly in their capacity to sensitize mice, with Ara h1 inducing the strongest IgE response. Interestingly, as demonstrated previously [55], depending on the route of provocation, peanut proteins differed even more in their capacity to cause allergic effector responses, such as mast cell degranulation and systemic anaphylaxis. For instance, only Ara h2 and 6 were able to elicit mast cell degranulation after oral challenge. In future studies, the mechanism behind the functional differences of individual peanut allergens and their cross-reactivity on a T cell and antibody level will be investigated.

Both *in vitro* and *in vivo* exposures to allergens in the context of CT reveal the role of intestinal epithelial cells, IELs, and DCs during allergic sensitization. In addition, these findings are relevant to risk assessment of novel proteins and illustrate the usefulness of mouse food allergy models to examine sensitization and effector responses to potential allergens at different levels in the allergic cascade both *in vivo* and *in vitro*.

### Tools and technologies for immunogenicity and allergenicity risk management

Introduction of novel protein content has enabled major advances in the development of a broad range of consumer products, including foods. However, as these products reach the market, entire populations will be exposed to the novel proteins they contain. Unwanted immune responses to novel proteins have the potential to cause serious health problems, as new food allergies could develop. Managing the risk of allergenicity in protein development is a complex, bioanalytical challenge to address. A range of technologies for assessing protein sensitizing potential have been developed. The aim was to provide tools that could be used in the earliest stages of product (ingredient, additive, or biologic) development so that the results obtained could inform future work. The reasoning was that this would enable a faster, more-cost effective transition from bench to market. Where products would have to undergo regulatory approval, the aim was to provide robust supporting data.

As an example, Humira® (adalimumab), an anti-TNFα therapeutic antibody employed in autoimmune disease indications, was investigated. It has been reported in prospective clinical studies that 28% of patients receiving the drug develop anti-adalimumab antibodies [56] that rendered the treatment ineffective. The development of these anti-drug antibodies is attributable to the presence of immunogenic T-cell epitope content within adalimumab. The technologies developed by the investigators allowed the investigation, characterization, and measurement of the impact of this immunogenicity, and their work with adalimumab suggests how the same methodologies could be applied to any novel protein to assess the likelihood of allergic responses occurring *in vivo*. Using blood samples from healthy donors, investigators measured peptide presentation by HLA-DR with their mass spectrometry-based ProPresent™ assay. To confirm these findings, the binding of the identified peptides was analysed in a physical HLA-peptide binding assay. This assay, the ProImmune REVEAL® MHC-peptide binding assay, compares the amount of peptide-associated MHC present for different combinations of peptide/MHC. An array of different HLA molecules was tested, covering over 90% of the global population, so the potential impact of neo-epitopes across large populations was assessed.

Measurement of CD4^+^ T-cell proliferation in response to autologous DCs loaded with test peptides is another assay optimized for immunogenicity testing. Using these CD4^+^ T-cell proliferation assays with an adalimumab-derived peptide library, it was observed that HLA-DRB1*01:01-positive donors made responses to several of the epitopes identified using ProPresent™, providing further support for the idea that these epitopes contribute to the immunogenicity observed in patients. The innovations made in investigating the potential sensitizing properties of novel proteins at an early stage of their development hold great promise for the future of allergenicity risk management. Used to inform work on novel proteins, these techniques could contribute to a future in which allergenic novel proteins rarely, if ever, enter the food chain.

### The BALB/c mouse model of allergy for the assessment of sensitizing properties of proteins and foods and their alteration by environmental conditions

The assessment of the allergy risk of a novel protein mainly focuses on its potential to elicit an allergic reaction in consumers already sensitized to a cross-reacting protein. However, reliable tests are missing to definitively predict on their own the potential of novel proteins to sensitize *de novo* atopic individuals. Such potential depends on intrinsic characteristics of the protein (*e.g.*, structure, function, and physicochemical properties) but also on complex interactions between the genetic background and physiology of the consumers and environmental conditions. The impact of extrinsic factors such as the composition of food matrix, food processing, as well as the dose, route, and frequency of exposure, is of major importance to modulate the potential of a protein to induce sensitization versus tolerance.

Allergic sensitization to food proteins is considered to result from an impaired development of oral tolerance or a breakdown in an existing oral tolerance. In this regard, integrated animal models such as the BALB/c mouse, a Th2 biased responder strain, may provide useful information. Currently, such models are not intended to be used as routine tests to predict the risk of predisposed humans to develop an allergic reaction in everyday life conditions, but rather, they are designed to compare the sensitizing properties of allergenic proteins in different conditions. They also allow a better understanding of the mechanisms that polarize and modulate the immune response after exposure to a protein inducing either oral tolerance or allergic sensitization.

Experimental sensitization to purified proteins or whole foods, *i.e.*, cow’s milk (CM) or peanut, is achieved by intraperitoneal (ip) or intragastric (ig) administration in the presence of Th2 adjuvants, namely alum, incomplete Freund adjuvant (IFA), or CT. Sensitization is qualitatively and quantitatively assessed by analysis of the intensity and specificity of the antibody (*i.e.*, IgE, IgG1, and IgG2a) responses to the different proteins of the food and to the different epitopes of each purified allergens. Cytokines produced after reactivation of spleen cells are also evaluated. Sensitization is further confirmed by the analysis of the early phase of the reaction (*e.g.*, leucotrienes, prostaglandins, histamine production) and of the mediators and biomarkers (cytokines, eosinophilia) and symptoms of the late phase of the allergic reaction after experimental elicitation of sensitized animals.

Based on results, BALB/c mice can display similar reactions as allergic humans. Ip or ig sensitization with whole CM or purified CM proteins (CMP), *e.g.*, ß lactoglobulin (BLG), results in the production of IgE antibodies specific to the same CMPs as in allergic humans and to the same epitopes on BLG as those identified in humans [57]. However, the responses may vary greatly depending on both intrinsic and extrinsic factors.

#### Influence of the protein’s structure

The structure of BLG used for sensitization and elicitation, *i.e.*, native (where both linear and conformational epitopes are present) versus denatured (where only linear epitopes are available), influences the manifestation appearing during the active phase of the allergic reaction. Two different mechanisms of mast cell activation, of different intensity, may be triggered. They specifically involve either peptido-leukotrienes in case of challenge by native BLG or prostaglandin D2 in case of challenge by denatured BLG [58].

#### Influence of the dose of exposure: expression of sensitizing versus tolerizing properties of BLG

Oral administration of raw CM in appropriate conditions, without adjuvant, to mice that have been previously ig-sensitized to BLG may only trigger mild clinical reactions but further enhances sensitization to BLG. It also results in sensitization against other CMPs such as α lactalbumin, casein, and lactoferrin that would have not sensitized naïve mice in similar conditions of administration. In contrast, a systemic tolerance can be totally or partially induced by administration of BLG without adjuvant via the ig route. It prevents further sensitization to BLG but also to other CMPs after ig administration of pure BLG or CM in the presence of Th2 adjuvants. Therefore, depending on the conditions of exposure, BLG may act either as a sensitizing or a tolerogenic protein. The underlying mechanisms are likely to be either 1) an increase of the transcellular and paracellular gut epithelial permeability and development of a local allergic reaction leading to Th2-cell differentiation and specific response to bystander proteins, or 2) the activation of BLG-specific T regulatory cells that results in induction of a pro Treg gut mucosal microenvironment [59].

#### Influence of the route of administration and the microbial environment of the allergen

Brief cutaneous exposure to peanut protein extract via applications on intact skin potentiates the gastrointestinal sensitizing properties of peanut allergens after subsequent ig administration in the presence of CT. However, this impact on sensitizing potency is much decreased when skin applications are made in the presence of microbial immunostimulatory (*e.g.*, CpG) sequences [60]. The influence of the microbial environment of the protein is also demonstrated when using lactic acid bacteria (LAB) as a vector for *in situ* delivery of the allergen. Pretreatment of mice by oral administration of recombinant LAB producing BLG, before ip sensitization to BLG in the presence of IFA, results in a decrease of anti-BLG IgG1 antibody response, a decrease of IL5, and increase of IFNγ production by reactivated spleen cells, and thus prevents the mice from a subsequent sensitization by induction of a moderate Th1-type response counterbalancing the Th2 one. Administration of non-recombinant LAB did not demonstrate a significant preventive effect [61].

#### Influence of the gut microbiota

The gut microbiota interacts with the host, the food, and the environment (including the microbial environment). It takes part in the digestive processes of the food proteins and also acts on gut mucosal permeability and on the stimulation and maturation of the gut immune system. The influence of the gut microbiota on the sensitizing property of a food protein has been studied on germ free (GF) versus conventional BALB/c mice. Effective sensitization can be achieved with production of specific IgE antibodies and secretion of IL 5 by spleen cells after ip administration of BLG in the absence of adjuvant to GF BALB/c mice, whereas no such response is observed in conventional mice. After ip sensitization in the presence of IFA, the Th2 response is earlier, more intense, and more persistent in GF than in conventional mice [62].

It can be concluded that sensitizing properties of proteins result from intrinsic, structural, and physicochemical properties of the protein that interact with the host genetics and physiology and with environmental conditions. To study such interactions, including the role of the gut microbiota which is at the interface of those factors, the BALB/c mouse provides a useful integrative model, which may reproduce some aspects of the situations observed in allergic humans and allows the investigation of the underlying mechanisms of allergy development. Among others, extrinsic factors such as the dose, route, and mode of administration, but also the presentation and the environment of the protein, influence the polarization of the immune response toward a Th1-, Th2-, Th17-, or a Treg-type response and its intensity, and thus modulate the sensitizing potency of proteins leading either to oral tolerance or to allergic reaction.

### The Brown Norway rat model to assess the oral sensitizing properties of food proteins

Animal models to study the sensitizing potential of new proteins should satisfy several important criteria [63,64]. These criteria are not easy to meet all in one model. Selection of species and strain, age, route, and dose of exposure for sensitization and challenge, as well as the use of adjuvants, are important criteria to consider. For the evaluation of the intrinsic potential of new food proteins to induce an allergic sensitization, oral application was preferred in the studies. An oral sensitization protocol using Brown Norway (BN) rats was developed based on comparative sensitization studies performed using different strains of rats [65]. Daily ig administration of 1 mg OVA during 42 days, without the use of adjuvants, was found to be the most optimal protocol [66]. It resulted in OVA-specific IgG, as well as OVA-specific IgE, responses in almost all rats (80%) as measured by ELISA and passive cutaneous anaphylaxis (PCA). The absence of detectable OVA-specific IgE responses in some rats was hypothesized to result from tolerance development due to dietary pre-exposure of the rats to OVA. Subsequent studies indeed supported that unintended dietary pre-exposure of the rats or their parental generations to soy [67] or peanut [68] seriously reduced oral sensitization with these respective food proteins or cross-reacting proteins (*e.g.*, dietary elimination of soy in case of sensitization to peanut proteins). In the BN rat food allergy model, oral challenge reactions were also investigated [69]. In OVA-sensitized BN rats, it was shown that on an oral challenge with OVA, gut permeability was increased as evidenced by an increased uptake of a bystander protein (α-LG). However, no clear systemic effects on respiratory functions (*e.g.*, decreased respiratory frequency) and systolic blood pressure were observed, as only a minority of the challenged animals were affected. Although under the performed test conditions, the oral challenge with OVA induced only minor effects on the respiratory system or blood pressure in a minority of animals, this low incidence is considered to be in accordance with clinical practice in food allergic patients. In subsequent studies with BN rats, the sensitizing potential of hen's egg white (HEW), CM, [70] and crude raw and roasted peanut extracts were studied [71]. Although antigen-specific IgG responses were found on daily gavage dosing of the animals to different concentrations of HEW or CM, only a limited number of IgE responders were observed as measured by PCA. However, immunoblotting experiments with these rat sera demonstrated specific-IgE antibodies against both HEW-proteins and CM-proteins [70]. Moreover, both IgG and IgE antibodies present in sera of orally sensitized rats to HEW or CM and in sera of HEW- or CM-allergic patients recognized a comparable profile of allergens in these food products. These results indicate that the specific protein recognition of induced antibodies in the BN rat is comparable to that observed in sera from allergic patients [70]. In respect to the sensitizing potency of crude-raw and roasted peanut protein extracts, as measured by the Th2-mediated IgG2a production, no clear difference was observed, and only a limited number of rats were IgE positive as measured by PCA. A remarkable observation was made when the IgG2a responses of orally and intraperitoneally sensitized animals with peanut proteins were measured against the three purified major peanut allergens Arah1, Arah2, and Arah3. After oral sensitization, IgG2a antibodies were directed against all three major peanut allergens, whereas after ip sensitization IgG2a antibodies were mainly directed towards Arah2 [68,71].

The allergenicity of several purified weak- (Sol t1 from potato), strong- (Ara h1 from peanut and Pen a1 from shrimp), and non-allergenic proteins (beef tropomyosin) were studied in the BN rat on oral and ip application at different dose levels and with or without the use of adjuvant [72]. The most robust responses on oral dosing were observed with the highest protein dose tested (1 mg/kg/day for 42 days), and the use of an oral adjuvant (cholera toxin) did not increase the sensitivity of the model. The prevalence of positive IgE antigen-specific ELISA results from the orally sensitized rats was similar to the prevalence expected from the human population (based on challenge reactions to these proteins), with Ara h1 > Pen a1 > Sol t1 and beef tropomyosin being negative. The experiments to assess the relative allergenicity of the various purified proteins were conducted in duplicate, and remarkable differences were observed in response in these two experiments, which were probably due to differences in genetics, diets, and other factors. The animals used in these studies were obtained from two different suppliers and raised on different diets (both containing soybean, corn, and wheat as principal components, and one diet also containing fish meal). The results obtained emphasize again the importance of dietary control and genetic differences of the animals when oral sensitization studies need to be performed. In a separate unpublished study (Knippels, Koppelman and Penninks, personal communication), the allergenicity of the potent allergen from Brazil nut, *e.g.*, 2S albumin (Ber e1), and a reduced and alkylated 2S albumin (RA-2S albumin, the loss of disulfide bridges resulted in reduced digestive stability) were tested in the BN rat model. On daily gavage administration of 1 mg/kg for 42 days, 70% of the animals developed Ber e1-specific IgG antibodies, and 50% of the animals developed Ber e1-specific IgE antibodies. The animals sensitized with the RA-2S albumin only developed IgG antibodies and no RA-2S albumin specific IgE.

In conclusion, the results obtained to date indicate that the BN rat may be a useful and predictive animal model to study the potential oral allergenicity of “novel” food proteins. However, additional testing with either whole foods or with purified non-, weak-, and strong-allergenic proteins are needed to further validate the BN rat model. In addition, special attention should be given to the animal diets, which should be free of the test protein (or cross-reacting proteins with the test protein) for at least two generations in order to prevent the development of oral tolerance to the test protein.

### Limitations and possibilities of animal models for human allergenic risk evaluation

There are many unanswered questions relating to food allergy sensitization in humans. It is not known i) in what situations sensitization takes place, *i.e.*, contribution of different routes (oral, dermal, respiratory); ii) dose-response relationships; iii) influence of frequency of exposure; iv) role of digestion; v) role of infection; and vi) by-stander effects of other allergens. In addition, it is not known under what conditions oral tolerance develops. With all these unanswered questions, it is a significant challenge to develop an animal model that, with relatively few animals, is able to predict if a protein is not only a potential allergen but also can predict its potency, a prerequisite for risk evaluation.

One of the pitfalls may be the premise that an animal model needs to mimic the disease. Chemical contact sensitizers may be predicted in an animal test, the Local Lymph Node Assay (LLNA) [73]. This assay is based on detailed mechanistic knowledge of contact sensitization, including knowledge of the dose-response relationship. The outcome of the test is sensitization measured as cell proliferation in the regional lymph node. Here the endpoint is not clinical symptoms but biomarkers of sensitization, and the design is based on detailed knowledge about the mechanisms behind contact sensitization.

As apparent from the above discussion, there is a lack of detailed knowledge on the mechanisms leading to food allergy enabling the design of a mechanism-based food allergy test. Nevertheless, animal models are used in food allergy research to understand food allergens and food allergy sensitization. In general, two sensitization protocols are used: ip or oral dosing regimens with or without the use of adjuvant. Adjuvants may increase the immune response for both ip and oral immunizations, but are generally not needed for ip studies. While the use of cholera toxin is a prerequisite for inducing a specific IgE response in oral mouse models, BN rats may be used for oral studies without the use of adjuvant. How or if adjuvants change the functionality of the antibody response is not well described.

Depending on the questions needing to be answered, different sensitization protocols may be used. If one wants to know if an unknown protein is a potential food allergen sensitizing via the gastrointestinal tract, it is important to use a model that includes the GI tract. It is also important not only to measure IgE, but also to measure the functionality of the IgE response. This can be done *in vitro* measuring degranulation of rat basophilic leukemia cells (RBL assay) or *in vivo* by PCA. It is mandatory that the animals are on a diet free from the protein under investigation or cross-reacting allergens to get a valid answer [74].

**Figure 4:**
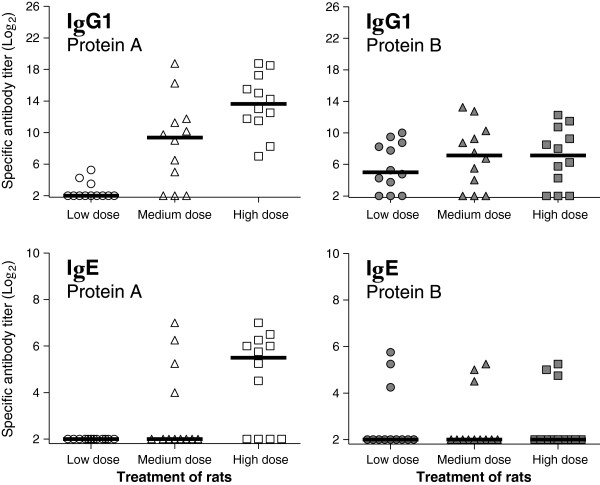
Model example: specific IgG1 and IgE measured by direct and capture ELISA, respectively, after oral doing of Brown Norway rats for 35 days without adjuvant with two related proteins.

The lack of dose-response knowledge in food allergy makes it difficult to interpret the results in a risk assessment context. If a protein in food induces a specific functional IgE response by the oral route, one can conclude that there is a hazard connected to the given protein. However, this does not provide information about the dose-response relationship. Most food allergens are major constituents of their “parent” food, but as the relationship between dose and sensitization (the shape of the dose-response curve) is unknown, this knowledge is difficult to include in the risk assessment. It is currently not possible to include dose-response relationships in the interpretation of sensitization studies, and it is, therefore, not possible to characterize the risk. Figure 4 shows a model example, the results of oral dosing of BN rats for 35 consecutive days with two related proteins. Protein A gives the highest immune response, both IgG1 and IgE, and the response is dose-related. With protein B there is no dose-response, but there is a response in the low dose. Which of these proteins is the strongest food allergen? With current knowledge, this question cannot be answered.

How then can/should animal models be used for human allergenic risk evaluation? Animal models in food allergy can be used to increase understanding of food allergens and food allergy sensitization, *e.g.*, the influence of digestion or processing or to compare closely related allergens. When using purified allergens it may be difficult to have a sufficient amount of allergen to perform oral studies. Intra-peritoneal dosing of BN rats has been used to compare the allergenicity of intact and digested Ara h 1, showing that digestion products of the 7S globulin Ara h 1 have similar allergenicity as the parent protein, being able to sensitize by the ip route and degranulate RBL cells [75]. Also, closely related 7S globulins from different plants have been compared. These proteins were readily digested and had similar digestion profiles. For these reasons, it is viewed as not optimal, but acceptable, to compare the intrinsic allergenicity of such purified proteins by the ip route. Investigators showed that the related 7S proteins induced an IgE response of the same magnitude, but the functionality of IgE induced by Ara h 1 was significantly higher compared to IgE induced by 7S globulins from pea, soy, and hazelnut [74]. Using the oral route makes it possible to study the influence of matrix and processing on allergenicity. Using oral dosing with peanut products, it has been shown that although extract of roasted peanuts is the most efficient elicitor of RBL cell degranulation, roasted peanut or peanut butter made from roasted peanuts are not better sensitizers than blanched (mildly heated) peanuts (unpublished observations).

In conclusion, the current lack of mechanistic knowledge of the sensitization process in food allergy makes it difficult to design and interpret predictive animal models. However, by using carefully designed studies, it is possible to use animal models to increase knowledge about the different factors influencing sensitization of known allergens.

### Conclusions: predicting allergenicity of new proteins

A number of foods containing GM crops have been introduced to the marketplace. These products have been carefully evaluated for their overall safety from an agronomic, environmental, performance, and equivalence perspective. Furthermore, studies have confirmed the safety of the GM crops and the novel protein(s) associated with the new trait(s).

One aspect of the novel protein safety evaluation is the question concerning their potential allergenicity. The allergenicity risk to consumers from GM crops may be placed into one of three categories.

The first risk category involves the transfer of a known allergen or cross-reacting allergen into a food crop. This risk is addressed by performing bioinformatics searches in order to show that the protein of interest does not match a known allergen [76].

The second risk category involves expression of proteins that may become *de novo* allergens. This risk has been studied by using various non-validated animal models, which have been developed over the past 15 years, including the well-known models described in Ladics et al. [72] and new experimental results observed in rat or mouse models described earlier in this paper. Although several promising tests have been proposed, none of them has been recognized as the ideal model for predicting the sensitization potential of proteins. In summary, the panel of scientific experts at the 2012 symposium agreed that no validated animal model that is predictive of protein allergenicity is currently available today.

The third risk category (though not within the scope of this paper) is the potential for increasing the allergenicity of an already allergic crop (*e.g.*, soybean) by increasing the expression of endogenous allergens.

In conclusion, the evaluation of protein allergenicity is currently based on a “weight-of-evidence” approach, which takes into account a variety of factors that have been associated with allergens, such as amino acid sequence identity to known allergens, stability to pepsin digestion or other enzymes *in vitro*, protein abundance in the crop, and the history of safe use of the source of the gene [20]. Animal models have been proposed for the safety assessment of novel proteins such as those expressed in GM crops; they may prove useful in the future but need further development and validation to improve their sensitivity and specificity.

## Discussion

GM food crops undergo an extensive safety assessment that has a record of producing safe consumer products. In fact, since the first GM crops were marketed in the mid-1990s, there have been no reports of any adverse effects associated with them. As part of the safety evaluation for GM crops, an allergenicity assessment is performed [20]. This assessment has several purposes: 1) to ensure that an existing allergen or cross-reactive protein(s) is not transferred into a new GM crop; 2) to demonstrate that a novel protein is unlikely to become a food allergen *de novo*; and 3) to evaluate existing endogenous allergen levels for potential increases in the new GM crop (*i.e.*, soybean) versus its non-GM control [77]. To help address the second issue, a number of investigators have examined the use of *in silico, in vitro* and *in vivo* methods to help predict the sensitization potential of novel proteins *de novo*. In particular, a promising process for assessing the risk of industrial proteins (*e.g.*, proteases) to cause respiratory sensitization in an occupational setting was described. This approach, however, is in the early stages of development and needs further evaluation with additional proteins. Likewise, *in vitro* models utilizing the co-culture of the MODE-K cell line or stem-cell derived intestinal organoids and IELs, or the ProImmune REVEAL® MHC-peptide binding assay on further development and validation, may hold promise for the future of allergenicity risk assessment. Some symposium participants have also been working on the development of models for predicting or ranking the potential allergenicity of food proteins in rats and mice that evaluate different endpoints, routes of exposure, and dosing regimens. A sensitive and specific validated animal model would help in the identification of proteins that might present an increased risk of sensitizing consumers if introduced in a GM food crop. While some progress has been reported with a limited number of proteins, none of the animal models that have been reviewed here have been thoroughly tested and validated with a wide range of allergens and non-allergens. To be considered predictive, the animal model should identify the allergenicity of a high percentage of known allergenic proteins and also predict low or no allergenic activity for the majority of currently consumed, low-, or non-allergenic proteins [72]. Even today, different mouse strains show different sensitization responses, which limit the ability to standardize a single model. While some models appear promising (*i.e.*, either *in silico*, *in vitro*, or *in vivo*), they need to be widely tested to be shown as predictive.

## Unpublished observations

Kroghsbo S, Rigby NM, Johnson P, Adel-Patient K, Bøgh KL, Salt LJ, Mills ENC, Madsen CB: **Roasting of peanuts does not increase sensitization. Examination of peanut products and native and processed Ara h 1 in Brown Norway rats (submitted)**.

## Competing interests

The views and opinions expressed in the paper are those of the authors, and do not necessarily reflect the views of the authors’ employers. The authors declare that they have no competing interests.

## Authors’ contributions

GSL, JF, RG, CH-G, KH-S, CBM, AP, AP, ELR, JS, and J-MW planned and organized the April 2012 Symposium on Sensitizing Properties of Proteins and contributed to this manuscript. All authors read and approved the final manuscript.
